# Coronavirus Disease 2019 Presenting as Abdominal Distension in a Mesothelioma Patient: Do We Need to Think Outside the Box?

**DOI:** 10.7759/cureus.9947

**Published:** 2020-08-22

**Authors:** Muhammad Irfan, Talal Almas, Irfan Ullah, Estelle Tran, Asim Ali

**Affiliations:** 1 Internal Medicine, Hayatabad Medical Complex, Peshawar, PAK; 2 Internal Medicine, Royal College of Surgeons in Ireland, Dublin, IRL; 3 Internal Medicine, Kabir Medical College, Peshawar, PAK; 4 Internal Medicine, Naseer Teaching Hospital, Peshawar, PAK

**Keywords:** atypical coronavirus, corona virus disease 2019, mesothelioma

## Abstract

The coronavirus disease 2019 (COVID-19) remains a major source of mortality globally. Although it usually presents with flu-like symptoms such as fever and sore throat, the intensity of the symptoms varies, constituting a spectrum ranging from asymptomatic to severe cases of acute respiratory failure. The highest proportion of severe cases occurs in older individuals and in those who have underlying health conditions and chronic comorbidities. We chronicle an atypical case of a 46-year-old male with stage IV mesothelioma who presented exclusively with complaints of acute abdominal pain and distension. Despite his cancer status, the patient’s respiratory functions remained unremarkable, accentuating the peculiarity of the case.

## Introduction

The coronavirus disease 2019 (COVID-19) is caused by the severe acute respiratory syndrome coronavirus 2 (SARS-CoV-2), a member of the Coronaviridae family. This novel pathogen belongs to the Betacoronavirus genus, which also includes the middle eastern respiratory syndrome coronavirus (MERS-CoV) [1]. Transmission occurs primarily through human-to-human contact via respiratory droplets. The virus travels through particles from the secretions of the infected individual and enters into direct contact with mucous membranes of the respiratory tract [1,2].

Infected individuals manifest a vast array of symptoms, ranging from asymptomatic to severe cases of respiratory compromise secondary to interstitial pneumonia and acute respiratory distress syndrome (ARDS) [1,2]. Pertinently, the majority of the reported cases present with mild symptoms, such as fever, cough, fatigue, sore throat, and loss of taste and smell [3]. Imperatively, some gastrointestinal disturbances, including diarrhea, vomiting, and nausea have been reported; however, in patients with a prior history of lung carcinoma, respiratory compromise remains the predominant complaint. Furthermore, increased mortality is noted in patients with chronic obstructive pulmonary disease (COPD) and pulmonary malignancies [3]. We hereby elucidate the case of a middle-aged male with mesothelioma who presented with complaints of acute abdominal pain and distension. In order to alleviate his symptoms, abdominal paracentesis was performed. Subsequent testing revealed an underlying COVID-19 infection. Intriguingly, despite a COVID-19 infection on a background of mesothelioma, no symptoms indicative of respiratory compromise were present.

## Case presentation

We delineate the case of a 46-year-old male who presented to the emergency department with chief complaints of acute abdominal pain and distention. His initial blood pressure was 113/81 mmHg (normal range = 90/60-120/80 mmHg), temperature was 98°F (normal range = 97-99°F), heart rate was 112 beats per minute (normal range = 60-100 beats per minute), respiratory rate was 19 breaths per minute (normal range = 12-20 breaths per minute) and oxygen saturation was 90% (normal range = 95-100%) without any oxygen support. On examination, his chest was clear bilaterally. Notably, his abdomen was distended and tensed due to ascites. The patient has a past history of stage IV mesothelioma as well as hepatitis C. He has been on chemotherapy for the last 15 months to alleviate his mesothelioma symptoms. 

**Figure 1:**
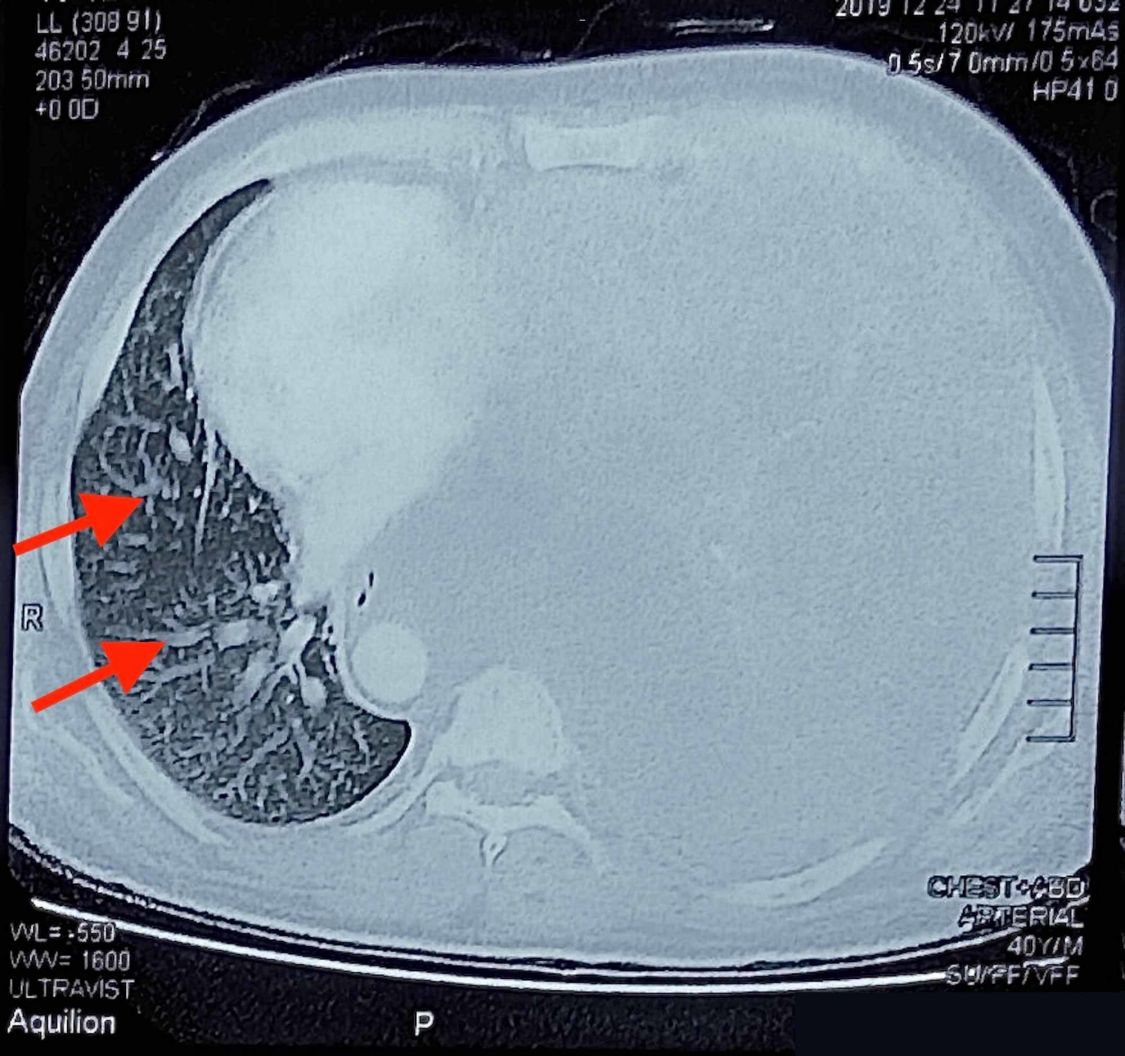
A chest CT showing a large lobulated circumferential mass filling the left hemithorax with multifocal extensions through diaphragm, chest wall and mediastinal fats. Enlarged lymph nodes in the left hilar, tracheobronchial and subcarinal regions are noted. Ground-glass opacities are also seen (red arrows). CT: computed tomography.

Due to his mesothelioma status, a COVID-19 polymerase chain reaction (PCR) was performed and turned out positive. To better elucidate his disease status, a chest computed tomography (CT) scan was performed and revealed ground-glass opacities (Figure 1). Additionally, a large lobulated circumferential mass filling the left hemithorax with multifocal extensions and hilar, tracheobronchial and subcarinal lymphadenopathy was also seen (Figure 1).

**Figure 2:**
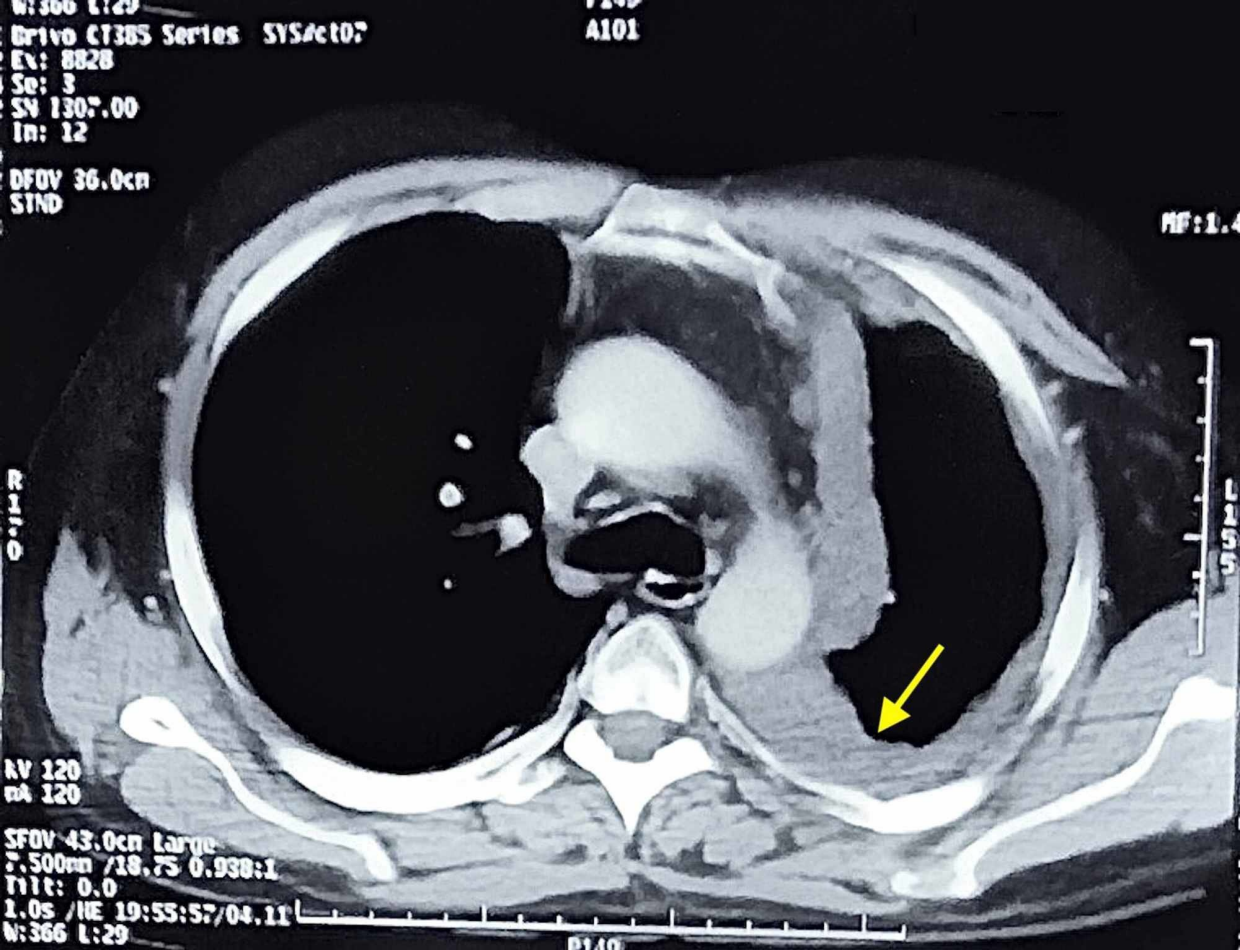
A chest CT showing residual circumferential hypodense pleural thickening involving the mediastinal, costal and diaphragmatic pleural surfaces. Erosion of the inner aspect of multiple ribs is noted. Left lung is inflated with fibroelastic bands (yellow arrow), indicative of stage IV mesothelioma. CT: computed tomography.

Furthermore, the chest CT scan divulged residual circumferential hypodense pleural thickening of the pleural surfaces (Figure 2). These findings were in concordance with a prior diagnosis of stage IV mesothelioma. 

Thereafter, the patient was moved to the isolation ward. Interestingly, while the patient complained of acute abdominal pain and heaviness, he manifested no symptoms suggestive of a COVID-19 induced respiratory compromise. Prior to this, the patient had been on regular follow-up with no exacerbations of his mesothelioma. For therapeutic purposes, an ascitic tap procedure (paracentesis) was performed, and the patient’s clinical symptoms promptly abated. Due to his COVID-19 and mesothelioma status, the patient was prescribed azithromycin (500 milligram (mg) once daily), hydroxychloroquine (200 mg twice daily), cetirizine (10 mg once daily), paracetamol (500 mg thrice daily), cosome (2 teaspoons full thrice daily) and aldactone (100 mg once daily). The patient remains well to date, with no need for ventilation or oxygenation.

## Discussion

The novel COVID-19 has proven to entail a worse prognosis with adverse outcomes in the elderly and those with underlying health conditions and comorbidities such as hypertension, obesity, diabetes, and cardiovascular pathologies [1-3]. Patients with impaired or compromised lung conditions, such as those with COPD, asthma, idiopathic pulmonary fibrosis, and cystic fibrosis, are at a higher risk of developing severe manifestations of this infective ailment. In one particular study, it was concluded that individuals with COPD who tested positive for COVID-19 manifested a higher risk of severity and COVID-19-related mortality [4].

COVID-19 continues to critically impact the lives of many vulnerable populations, including cancer patients. Malignancies are noted to render immune systems more vulnerable to infective ailments [4,5]. Additionally, anti-cancer treatments such as chemotherapy, radiation, and targeted drug therapies can further predispose cancer patients to the development of severe manifestations of COVID-19. Their immunosuppressed state predisposes them to respiratory pathogens and severe pneumonia. Pertinently, a study revealed that, in a sample of 28 cancer patients with a confirmed COVID-19 infection, 53.6% of the patients developed serious symptoms, 21.4% were admitted to the intensive care unit (ICU), 35.7% had life-threatening complications, and 28.6% died [4,5]. 

In patients with lung cancer, a rapid progression of COVID-19, as well as the development of serious anoxia, is often observed [5]. A plethora of studies have espoused the notion that a significant mortality rate is observed in individuals with lung cancer with COVID-19 compared to all COVID-19 patients, with a 52.3% mortality rate in the analyzed subgroup of cancer patients [6]. In addition to suboptimal pulmonary functions, most lung cancer patients are also on chemotherapy, which further enervates their immune systems. Liang et al. reported data on 18 cancer patients infected with SARS-CoV-2 who were on chemotherapy. Of note, all of the patients studied demonstrated adverse outcomes [7]. Moreover, symptoms of COVID-19 can often overlap with those of lung carcinomas, and can include cough, fatigue, dyspnoea, and fever, often obscuring an accurate diagnosis [7]. Additionally, mesothelioma, an aggressive malignancy, further predisposes COVID-19 patients to highly unfavorable outcomes. The presence of COVID-19 in such patients often leads to lung swelling, retention of fluid waste, and cellular growth in the lining of the air sacs. Moreover, COVID-19 is established to impact the respiratory lining of the lungs and thoracic region, frequently eliciting symptoms such as dyspnea and cough [8]. Even in remission, these cancer patients are at higher risk of complications secondary to a COVID-19 infection [8,9]. Nevertheless, respiratory complaints, such as those delineated above, remain ubiquitous in this patient population. Interestingly, in our case, the patient presented with no signs of respiratory compromise despite his cancer status. Further confounding this presentation was the presence of his acute abdominal symptoms. Medical literature remains largely bereft of such instances; there is thus an unmet need for studies with larger sample sizes to be conducted.

## Conclusions

COVID-19 is an infectious ailment that elicits a plethora of symptoms, the most characteristic of which is the acute respiratory distress syndrome. Medical literature is replete with studies detailing the increased likelihood of severe manifestations in patients predisposed to pulmonary compromise. Within this subset, patients with a prior diagnosis of a pulmonary carcinoma, such as a mesothelioma, constitute a significantly high-risk group. Nevertheless, presentation with gastrointestinal symptoms remains exceedingly rare. Physicians should therefore remain cognizant of the possibility of gastrointestinal symptoms secondary to a COVID-19 infection even in the absence of respiratory symptoms.
